# The Missing Pieces: The Role of Secretion Systems in *Campylobacter jejuni* Virulence

**DOI:** 10.3390/biom13010135

**Published:** 2023-01-09

**Authors:** Amber D. Gabbert, Jennifer L. Mydosh, Prabhat K. Talukdar, Lisa M. Gloss, Jason E. McDermott, Kerry K. Cooper, Geremy C. Clair, Michael E. Konkel

**Affiliations:** 1 School of Molecular Biosciences, College of Veterinary Sciences, Washington State University, Pullman, WA 99164, USA; 2 School of Animal and Comparative Biomedical Sciences, The University of Arizona, Tucson, AZ 85721, USA; 3 Integrative Omics, Pacific Northwest National Laboratory, Richland, WA 99354, USA

**Keywords:** bacteria–host cell interactions, secretion, type III secretion system, type IV secretion system, type VI secretion system, effectors

## Abstract

*Campylobacter jejuni* is likely the most common bacterial cause of gastroenteritis worldwide, responsible for millions of cases of inflammatory diarrhea characterized by severe abdominal cramps and blood in the stool. Further, *C. jejuni* infections are associated with post-infection sequelae in developed countries and malnutrition and growth-stunting in low- and middle-income countries. Despite the increasing prevalence of the disease, campylobacteriosis, and the recognition that this pathogen is a serious health threat, our understanding of *C. jejuni* pathogenesis remains incomplete. In this review, we focus on the *Campylobacter* secretion systems proposed to contribute to host-cell interactions and survival in the host. Moreover, we have applied a genomics approach to defining the structural and mechanistic features of *C. jejuni* type III, IV, and VI secretion systems. Special attention is focused on the flagellar type III secretion system and the prediction of putative effectors, given that the proteins exported via this system are essential for host cell invasion and the inflammatory response. We conclude that *C. jejuni* does not possess a type IV secretion system and relies on the type III and type VI secretion systems to establish a niche and potentiate disease.

## 1. Introduction

*Campylobacter*, a genus of Gram-negative, zoonotic enteropathogens, is the leading bacterial cause of gastroenteritis worldwide. Of the known 31 *Campylobacter* species, *Campylobacter jejuni* is primarily responsible for human *Campylobacter* infection, with *Campylobacter coli* being a minor contributor [1]. Poultry is the major source of *Campylobacter* infections in humans, and the most common symptom of *C. jejuni* infection is the acute onset of diarrhea. *C. jejuni* virulence is dependent on bacterial motility and the secretion of a range of proteins. Unlike other Gram-negative bacteria, *C. jejuni* harbors only a few designated secretion systems that transfer bacterial proteins to the eukaryotic host cell. In this review, we focus on the *Campylobacter* secretion systems that contribute to host-cell interactions and pathogen survival within the host. We emphasize the type III secretion system (T3SS), which has been shown unequivocally to play a role in the pathogenic invasion of epithelial cells of the human intestine. This review begins with an overview of *C. jejuni* pathogenesis and the increasing public health concern in both developed and low-resource countries, followed by the emergence of antimicrobial-resistant isolates of *Campylobacter* over the past decade.

## 2. Ecology and Clinical Manifestation of *C. jejuni* Infection

Each year, campylobacteriosis, the disease caused by *C. jejuni*, causes approximately 550 million cases of gastrointestinal (GI) disease worldwide, including approximately 220 million cases in children under the age of 5 [2,3,4]. In the United States, an estimated 20 out of 100,000 people are diagnosed with *Campylobacter* enteritis per year, according to the Foodborne Diseases Active Surveillance Network [5]. Campylobacteriosis is one of the most common causes of hospitalization for gastroenteritis and GI disease-related complications [3]. Over the past decade, the frequency of disease outbreaks has continued to rise worldwide. Recent outbreaks in developed countries have been linked to *Campylobacter* isolates with extensive antimicrobial resistance [6]. However, the true magnitude of the impact of *Campylobacter* on human health is poorly understood. Studies have estimated that 11% of campylobacteriosis cases are reported in the United Kingdom, 8% are reported in the Netherlands, and only 3% are reported in the United States [7]. Reporting of cases is known to be even lower in low-resource countries.

In industrialized countries, the primary transmission source of *C. jejuni* is associated with the handling and consumption of poultry. Chickens are a commensal host of *Campylobacter* species, namely *C. jejuni*; the intestinal tracts of chickens are endemically colonized by 2 to 4 weeks of age [8,9]. After initial ingestion, *C. jejuni* travels through the intestinal tract to the cecum, and colonization of an individual chick can occur within 24 h following exposure to as little as 35 colony-forming units (CFU) of *C. jejuni*. Upon encountering the mucosal layer of cecal crypts, the bacteria proliferate, resulting in colonization levels of 10^6^ to 10^8^ CFU/g of cecal content. Subsequently, within several days, greater than 95% of a flock will become colonized. Several phenotypes vary between *C. jejuni* isolates that will impact colonization potential, including motility, chemotaxis, and, most importantly for this review, flagellar function [10].

### 2.1. Ecology and Transmission Dynamics

**Figure 1:**
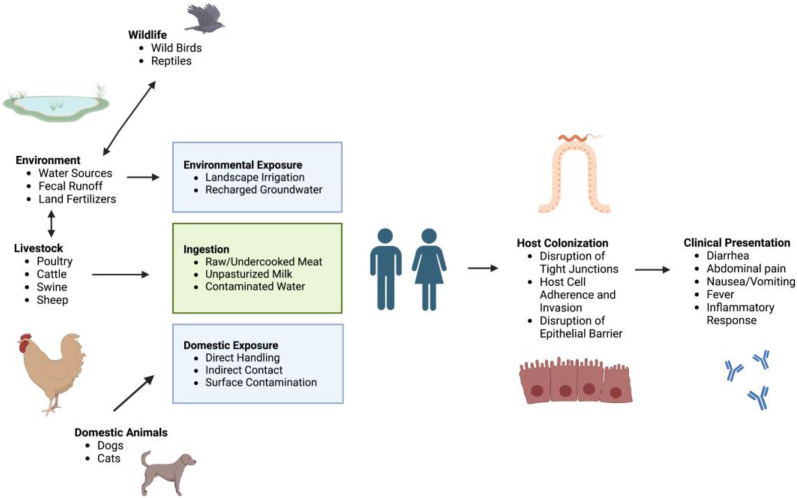
Schematic diagram demonstrating the relationship between the ecological dynamics and transmission of *C. jejuni*. Contamination of environmental resources by colonized hosts linked to cross-contamination of agriculture products, steel surfaces, and water sources. Ingestion of contaminated food products, direct handling of colonized animals, and accidental contact with contaminated environmental resources lead to human exposure to *C. jejuni* (left). After colonizing its host, *C. jejuni* adheres to the cell surface of the epithelial wall, invades the cells, and disrupts the epithelial barrier. Cellular invasion provokes an inflammatory response, which leads to the development of diarrheal symptoms. Illustration created using BioRender.

As highlighted in Figure 1, the interfaces in the human–animal ecosystem pose moderate health risk concerns because of cross-contamination [7]. In broiler chickens and cattle, *C. jejuni* is the predominant colonizing species, whereas *C. coli* predominates in swine [11]. Animals can be exposed to *Campylobacter* by various routes, including a contaminated water supply and feces, leading to colonization of the distal intestinal tract. Contamination of the food chain by *Campylobacter* and transmission to humans is then facilitated by processing operations, such as evisceration, scalding, and defeathering [12]. Contaminated processing water used during the immersion-chilling steps results in the entrapment of bacteria within the skin of the carcass, which protects the pathogen from bactericides [12].

Additionally, stainless steel equipment in husbandry settings can harbor biofilms containing *C. jejuni* [13]. Studies have demonstrated that *C. jejuni* can directly adhere to stainless steel surfaces, allowing for the recontamination of poultry or other agricultural animals, even following sanitary practices [13]. *C. jejuni* can remain attached to surfaces under low-nutrient conditions and retain pathogenicity at 20 °C, suggesting that poultry processing equipment may harbor pathogenic *C. jejuni* in evisceration areas [13]. Moreover, recent studies have demonstrated high levels of *Campylobacter* contamination of poultry transportation materials, such as shipping crates, even after the cleaning and disinfection processes [14].

Human exposure to *Campylobacter* is often caused by the consumption of undercooked or under-processed foods or through contamination of food products during processing [9]. Dairy farms, however, also play a role in human transmission. Consumption of unpasteurized milk has led to sporadic outbreaks of *Campylobacter* infection internationally. A 2011 study in Canada estimated that about 9.2% of cases of campylobacteriosis were caused by the consumption of raw milk [15]. In slaughtered beef cattle, *Campylobacter* is primarily isolated from the large intestine, gall bladder, and small intestine. Cattle raised in feedlots, compared to pastures, are more likely to be colonized by *Campylobacter* [16]. Humans may be exposed to *Campylobacter* from cattle by consumption of undercooked beef or through accidental contact with fecal runoff [17].

Domestic animals can also carry *Campylobacter* bacteria that can spread to people and cause illness. Recent outbreaks in the United States have been linked to pet store puppies. Investigations of a puppy-exposure outbreak from 2016–2020 showed that the outbreak isolates of *Campylobacter* were resistant to all common antibiotic treatments used to treat campylobacteriosis, such as macrolides and quinolones [18], likely due to ~95% of the puppies having been treated with at least one dose of antibiotics.

In addition to agricultural and domestic animals, environmental factors, such as water supplies and wildlife, can also lead to *Campylobacter* exposure. Humans may be exposed through water systems by accidental ingestion, following landscape irrigation, or by consumption of recharged groundwater [19]. Although the ecological significance of wild bird transmission to humans has not been fully characterized, *C. jejuni* is the most common species isolated from wild birds and is therefore considered a possible source of transmission [20].

### 2.2. Clinical Presentation and Medical Complications of Campylobacteriosis

**Figure 2:**
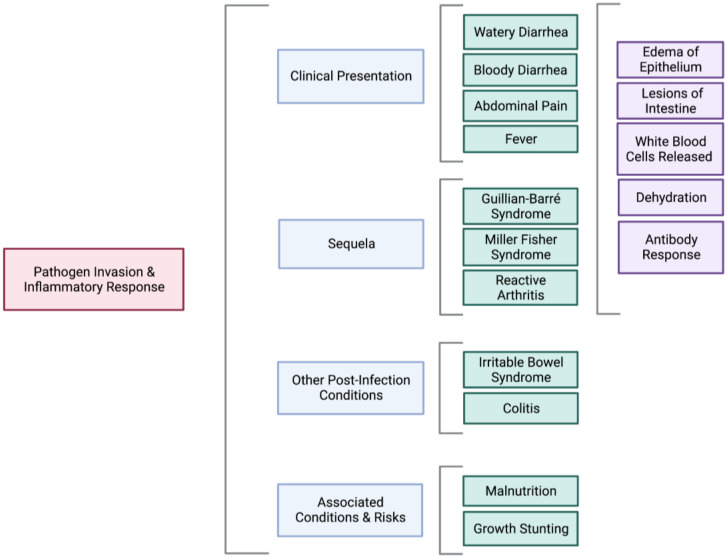
Clinical outcomes associated with *C. jejuni* infection. The primary clinical features of *Campylobacter* infection are diarrhea, abdominal pain, and transient fever. These symptoms develop from the pro-inflammatory response induced by pathogen invasion. Although rare, post-infection sequela such as Guillain-Barré syndrome (GBS), Miller-Fisher syndrome, and reactive arthritis may arise due induction of an antibody response. Other pre-existing conditions, such as irritable bowel syndrome (IBS) and colitis, might be exacerbated by *C. jejuni* infection. In low-resource setting countries, *Campylobacter* infection is correlated with malnutrition and growth stunting, though the underlying mechanism remains unclear.

The most common clinical presentation of *Campylobacter* enteritis in industrialized countries is diarrhea, typically accompanied by increased leukocytes or blood cells in stools (Figure 2) [2,3,4]. For adults with diarrheal illness, *C. jejuni* is the most frequently isolated species subtype in stool cultures but is largely absent in asymptomatic and otherwise healthy individuals (less than 1.3%) [2,21]. Similar results were observed in a study of 29 household contacts of individuals with diarrhea secondary to *Campylobacter* infection [21]; 7 of 11 household contacts who reported having diarrhea had positive stool cultures, whereas only 1 of the 18 asymptomatic contacts had positive stool cultures. These data indicate the infrequent isolation of *C. jejuni* from asymptomatic adults.

Additional clinical features associated with campylobacteriosis include transient fever, abdominal pain, cramping, nausea, and vomiting [4,22]. In some cases, individuals experience severe abdominal pain that clinically presents in a manner similar to a small bowel perforation [23]. Individuals have also reported fecal incontinence [23]. Symptoms usually begin 2–5 days following initial exposure and last for approximately 1–2 weeks [5]. The onset of fever in early infection typically resolves after 24 h, though occasionally, it lasts for a few days [23]. Most cases of campylobacteriosis are mild, where symptoms resolve without medical intervention. However, antibiotic treatment may be necessary for more severe cases of campylobacteriosis [2].

*Campylobacter* infection may cause bacteremia in individuals that are immunocompromised or with an underlying disease [24,25], such as carcinoma or liver disease [26]. The incidence of *Campylobacter* bacteremia is approximately 1.5 cases out of 1000 *Campylobacter* infections, though it varies among age distributions [27]. The incidence in individuals over the age of 65 is about 5.9 per 1000 *Campylobacter* infections, whereas children from ages 1–4 have an incidence rate of 0.3 per 1000 [27]. The species most commonly recovered from systemic infections is *Campylobacter fetus* [28]. *Campylobacter* bacteremia may lead to tissue localization, most notably in the vascular endothelium [28].

In low-income and low-resource countries, infants ages 0 to 12 months have increased *Campylobacter* prevalence, with positive samples being found for both normal and diarrheal stools [29]. Children in rural communities tend to have a frequent diarrheal illness with a stool positive for *Campylobacter* compared to children in urban communities [30]. *C. jejuni* is the most identified species, followed by *C. coli* [30]. The high prevalence in infants ages 6 to 12 months in low-income countries is also associated with the growth stunting [29]. The mechanism resulting in reduced growth due to *Campylobacter* infection remains unclear, but it is thought to be related to an inflammatory response [29].

### 2.3. Guillain-Barré Syndrome and Other Autoimmune Disorders Causally Linked to C. jejuni Infection

Although uncommon, post-campylobacteriosis infection complications do arise, such as Guillain-Barré syndrome (GBS), Miller-Fisher syndrome (MFS), and reactive arthritis.

GBS has been the leading cause of flaccid paralysis since the eradication of poliovirus. GBS is a severe form of demyelinating polyneuropathy induced by an acute inflammatory response [31]. Approximately 50–75% of GBS cases are preceded by a bacterial or viral infection. Moreover, 10–30% of antecedent infections are related to GI or diarrheal illness, among which *C. jejuni* was identified as the most frequent trigger [32]. According to the Centers for Disease Control and Prevention (CDC), about 1 out of 1000 individuals develop GBS following a *Campylobacter* infection in the United States. Moreover, every 1 out of 8 individuals in the United States with GBS has had a recent *Campylobacter* infection [33]. MFS, a rare subset of GBS, is a neurological condition characterized by abnormal muscle coordination. Neuronal dysfunction of MFS is typically associated with the lower cranial and facial nerves (III, IV, and VI). However, there have been case reports relating to the involvement of the other cranial nerves [34]

GBS and MFS are sequelae for *C. jejuni* infection arising from the structural similarity of *C. jejuni* oligosaccharides to human gangliosides GM1 and GQ1b, thus provoking an autoimmune antibody response [35,36]. GBS cases that are preceded by campylobacteriosis exhibit more prolonged periods of disability and poorer outcomes [4]. One study found that patients who developed GBS and anti-GM 1 antibody, which were considered to have a preceding *C. jejuni* infection, had a more rapid progression of severe peripheral neuropathy compared to GBS patients without anti-GM1 antibodies [37]. Moreover, those individuals with GBS and anti-GM 1 antibody were less responsive to the plasmapheresis [37].

Campylobacteriosis has been linked to additional autoimmune disorders [38]. Some *C. jejuni* isolates have sialic acid lipooligosaccharides, a biomarker for reactive arthritis. Genotype studies showed that individuals infected by sialic-acid-positive *C. jejuni* isolates were more likely to have bloody diarrhea and a prolonged infection period [39], indicative of induction of an inflammatory response and release of white blood cells (WBCs). Similarly, these individuals have an elevated antibody response to *Campylobacter* infection [39], further suggesting an inflammatory response.

Another associated sequela following *Campylobacter* infection is post-infectious irritable bowel syndrome (PI-IBS). Prolonged changes in the small bowel microbiota may result from *C. jejuni* infection, such as reduced levels of *Methanobrevibacter smithii* [40], the predominant archaea methanogen in humans. The reduced load of *M. smithii* is a biomarker of GI diseases including IBS, as the *M. smithii* load is greater in healthy adults compared to individuals with chronic gastrointestinal illnesses [41]. Up to a year after the initial *Campylobacter* infection, individuals with PI-IBS may experience an acute increase in enteroendocrine cells, T lymphocytes, and gut permeability. The severity of diarrheal disease or length of initial illness does not correlate with the numbers of enteroendocrine cells or T lymphocytes [42]. The underlying mechanism of PI-IBS is poorly understood; however, it appears to involve immune response dysregulation that is not due to an antibody response [42].

### 2.4. Pro-Inflammatory and Immune Response

A hallmark of *Campylobacter* enteritis is an acute inflammatory response localized to the epithelium of the distal colon [43,44]. After the first few hours to days of *Campylobacter* infection, disruption of the tight junction protein claudin-1 leads to impairment of the epithelial barrier [45]. *Campylobacter* invasion activates host cell Toll-like receptors (TLRs), which respond to bacterial components such as DNA, lipoproteins, and lipopolysaccharides [46,47]. TLRs then promote the expression of adapter proteins that activate nuclear transcription factors, such as NF-κB, which in turn induce secretion of the pro-inflammatory cytokines, interferon-γ (IFNγ), tumor necrosis factor-α (TNFα), and interleukin-8 (IL-8) [45,48]. This pro-inflammatory response results in apoptosis of epithelial cells, edema of the surrounding tissue, and lesion induction [45]. Furthermore, TNFα assists in the expression of tight junction proteins and apoptosis [45]. Crypt abscesses may result from the migration of granulocytes, though the crypts remain structurally intact throughout the infectious process [43]. Clinically, bloody diarrhea is often the result of this pro-inflammatory signaling pathway [49].

Chemokine IL-8 is a chemoattractant that, when locally secreted, recruits polymorphonuclear leukocytes and neutrophils to the mucosal layer of the intestine [46,49]. The induction of IL-8 is dependent on the focal complex, which recruits focal adhesion kinase (FAK), paxillin, and epidermal growth factor receptor (EGFR). The cellular adhesion molecule β_1_-integrin interacts with FAK, which leads to phosphorylation and activation of FAK by the non-receptor tyrosine kinase Src. The FAK-Src complex subsequently phosphorylates paxillin, which recruits the mitogen-activated protein (MAP) kinase, Erk 1/2. Active Erk 1/2 translocates to the nucleus and activates the activator protein 1 (AP-1), which promotes the transcription of IL-8. Transcription of CXCL8, the gene encoding for IL-8, requires activation by both AP-1 and NF-κB [50].

### 2.5. Antimicrobial Resistance in C. jejuni

Treatment of mild campylobacteriosis cases in otherwise healthy adults typically involves the administration of antibiotics, rehydration, and clinical monitoring [4]. Common antibiotics used for the treatment of campylobacteriosis include ciprofloxacin and azithromycin; however, antimicrobial resistance to fluoroquinolones has been increasing over the past two decades [51].

The growing awareness of the antimicrobial resistance of *Campylobacter* has led to a greater demand for antimicrobial-free (AMF) farms, aka organic farms [52]. Studies have shown that *Campylobacter* prevalence is similar on both AMF and conventional farms for poultry and swine [52,53]. *Campylobacter* isolated from chickens raised on AMF farms were found to have greater resistance to erythromycin and tetracycline compared to conventionally raised chickens; whereas ciprofloxacin resistance was found to be greater in *Campylobacter* isolates from conventionally raised chickens [53]. Thus, the emerging issue of antimicrobial resistance persists in the absence of antimicrobial use in husbandry settings. 

During recent outbreaks related to pet store puppies, the outbreak strain XDR *C. jejuni* exhibited antimicrobial resistance to multiple classes of antibiotics, including macrolides, fluoroquinolones, lincosamides, ketolides, tetracyclines, and aminoglycosides [6]. One study found multidrug-resistant (MDR) isolates of *C. jejuni* to be susceptible to carbapenems, including imipenem/cilastatin [6]. Approximately 16% of reported cases of the 2016 outbreak in 17 states of the United States required hospitalization, which was thought to be due to the extensive antimicrobial resistance of the outbreak isolate [5].

It is important to note that *C. jejuni* has been reported to enter a persister state upon exposure to antibiotics such as penicillin [54] or ciprofloxacin [55]. Persister cells are phenotypic variants able to survive antibiotic treatment caused by reduced metabolic activity that prevents antibiotic function. The triggering mechanism for persister formation in *C. jejuni* remains to be fully elucidated, but it seems that stringent or SOS responses are not involved [55]. Rather, it appears that a fraction of the *C. jejuni* population enters this non-growing state because of metabolic dysregulation, causing a drop in energy levels [54], a mechanism first reported in *S. aureus* [56]. The persister phenotype has been reported to increase the tolerance of *C. jejuni* to fluoroquinolones. Antibiotic tolerance compromises the efficacy of antibiotic treatment by extending survival during antibiotic treatment and increasing the probability of mutations that confer antibiotic resistance [57]. The existence of persisters and antibiotic resistance in *C. jejuni* can explain, at least partially, the report of MDR isolates across the globe [58,59,60].

The clinical presentation of *C. jejuni* infection is intrinsically linked to an induced inflammatory response within the intestinal epithelium. *C. jejuni* virulence is multifactorial, requiring both intrinsic and exported bacterial components, some of which facilitate host cell interactions. In addition to the release of outer membrane vesicles (OMVs) [61], *C. jejuni* modulate the behavior of host cells via bacterial effector proteins exported by dedicated protein secretion systems. For example, once successfully colonized, *C. jejuni* can then secrete *Campylobacter* invasion antigen (Cia) proteins through a secretion system. In turn, these effector proteins activate signaling pathways that allow for cell invasion and evasion of host cell immune response. 

## 3. Secretion Systems in *C. jejuni*

Bacteria have evolved to possess several secretion systems to export macromolecules (small molecules, proteins, and DNA) to the environment and other cells. To date, six different secretion systems (type I to type VI) have been identified in Gram-negative bacteria. The number of *C. jejuni* genome sequences deposited in databases has significantly increased in the past decade, aiding in the analysis of specific virulence genes (genomic content) and comparison amongst isolates. Analysis of the isolates has enabled predictions of the core genes necessary for disease versus the variable (i.e., dispensable) genes, whose products contribute to *C. jejuni* disease. Based on the review of the literature, *C. jejuni* does not possess a type I or type V secretion system, and only a few genes have been identified that encode protein components found in the type II secretion system [62,63]. Here, we have applied a comparative genomic approach to assess the presence/absence of type III, IV, and VI secretion systems in *C. jejuni*. Moreover, in this article, we describe insights into the structural and mechanistic features of *C. jejuni* type III, IV, and VI secretion systems, as these systems have been proposed to contribute to the pathogenesis of this bacterium and have been the focus of investigations by multiple research laboratories.

## 4. Type III Secretion System in *C. jejuni*

The type III secretion system (T3SS) is the most complex and widely available secretion system among Gram-negative bacteria. This is commonly known as the ‘molecular syringe’ because of its structure and the release and transfer of effector proteins directly into the eukaryotic host cells. T3SS has some similarities to the bacterial flagellum in terms of its structure and shares some proteins that are homologous to the flagellum. A few Gram-negative bacteria (e.g., *C. jejuni*) that are motile do not have a dedicated T3SS; instead, the flagellum has a dual function (motility and protein export). Here, we discuss the differences in the T3SS between *C. jejuni* and *Salmonella* Typhimurium, two Gram-negative bacteria that cause gastroenteritis.

### 4.1. Comparison of Flagellar and Dedicated T3SS

**Figure 3:**
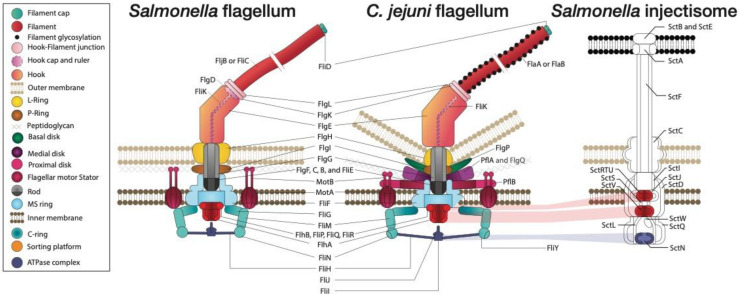
Comparison of the flagellum and injectisome of *Salmonella* to the flagellum of *C. jejuni*. The central panel depicts the *C. jejuni* flagellum based on current knowledge. *C. jejuni* proteins sharing significant homology with *Salmonella* flagellar proteins are indicated between the left panel, illustrating *Salmonella’s* flagellum, and the central panel. The flagellar proteins that do not share significant homologies or are unique to the bacterial isolate are individually annotated. The right panel shows the proteins of the *Salmonella* type III secretion system that share homology with the *C. jejuni* flagellar secretion system apparatus. The shading between the two panels indicates the existence of homologies.

The flagellum of *C. jejuni* is unique and presents many differences compared to other enteric pathogens that have a dedicated T3SS, such as *Salmonella* (Figure 3, Table S1). Unlike many enteric pathogens where flagella are numerous (6 to 10 flagella) and arranged peritrichously in a relatively flat part of the membrane [64], the two polar flagella of *C. jejuni* [65,66] are rooted in the cell membranes at the lower point of a downward concavity [65,67]. Whereas the outer membrane of *C. jejuni* is concave, where the flagella connect to the membrane, the inner membrane is relatively flat [67,68]. Blast analysis of the flagellar proteins of *C. jejuni* NCTC 11168 and *S.* Typhimurium strain 14028s showed that many protein components of the flagella did not have a homolog in the other bacteria (Figure 3). Even when homologs were found, the overall homologies were relatively low (median percentage of coverage = 89.0%, the median percentage of identity = 34.7%). In addition to the difference in the localization and composition of the flagellum, many differences and similarities were identified in the biogenesis and structure of their flagella. These will be discussed in the following paragraphs, from the tip to the innermost part of the flagella. 

The flagellar filaments of *C. jejuni* are composed of FlaA and FlaB, which share 95% sequence identity between these two proteins [69]. Filaments composed of FlaB only are shorter [70] and confer less motility [71,72]. However, FlaB filaments could help evade immunity [73] and confer resistance against flagellotropic bacteriophages [74]. In contrast, the filament of *Salmonella* is composed of one of the two flagellins FljB or FliC. Their production is autonomously regulated to produce a filament composed exclusively of one or the other flagellin. This filament variation is thought to be a mechanism to evade the host immune system by changing the flagellar antigenicity [75]. Whereas most bacterial flagellins have a conserved eight-amino acid epitope recognized by the Toll-like receptor 5 (TLR5), *C. jejuni* escapes TLR5 innate immune recognition by the mutation of this epitope [76]. Similar to other bacteria with polar flagella, *C. jejuni* flagellins are heavily O-glycosylated [77]. The nature of the glycosylation can vary in an isolate- or in a temporal-dependent fashion. The glycans harbored by flagellins can include pseudaminic acid [78], legionaminic acid [69], and their derivatives. Mutation of the flagellins at their different glycation sites results in reduced autoagglutination or virulence [77]. In *C. jejuni*, the glycans participate in the quaternary ultrastructure of the flagellar filament in the outer domains of the filament protein [76].

*C. jejuni* possesses a FliK homolog that seems to control the hook length similar to *Salmonella* FliK. However, the *C. jejuni* protein does not show strong homology with *Salmonella*’s FliK, and only a small domain near the C-terminus was reported to show a similarity to a conserved amino acid sequence from all known FliK sequences [79]. Nevertheless, because of its functional homology, the protein was named FliK. *C. jejuni* does not have the homolog of FlgD that serves as a scaffolding protein required for the hook assembly in *Salmonella* [80]. The main hook component, FlgE, has domains found only in *C. jejuni* and related bacteria (*C. coli* and *Helicobacter pylori*), making its hook about twice as large as that of *Salmonella* [81]. The hook structure of *C. jejuni* is divergent from *Salmonella*. In *Salmonella*, there are two proteins named FlgL and FlgK that form a smooth connection between the hook and the filament. *C. jejuni* possesses both FlgL and FlgK, but they are vastly different from their *Salmonella* counterparts both in sequence and structure [82]. Importantly, the export and delivery of some of *C. jejuni* virulence factors require the formation of a full hook complex comprising FlgE, FlgL, and FlgK [83].

*Salmonella* and *Campylobacter* possess homologous proteins that comprise the flagellar rod, including the L-ring (FlgH) and the P-ring (FlgI). Whereas the assembly of *Salmonella’s* P-ring requires the presence of a periplasmic flagellar chaperone, FlgA [84], no homolog of FlgA is present in *C. jejuni*, suggesting a different biogenesis of the P-ring. The flagellar rod is a drive shaft of the motor, transmitting motor torque to the hook and filament [85]. It is thought to be composed of homologous proteins in *Salmonella* and *Campylobacter*. In *C. jejuni*, one of these rod proteins, FlgG, is modified by a multi-substrate phosphoethanolamine (pEtN) transferase, EptC (*Cj0256*), that does not have a homolog in *Salmonella* [86]. Mutation of EptC results in decreased motility and reduced flagella production in *C. jejuni* [87].

Protein disks are found in the periplasm of bacteria with high torque polar flagella but not in peritrichously flagellated bacteria. *C. jejuni* has a basal disk attached to the P-ring located beneath the outer membrane. The basal disk is composed of subunits of FlgP [88]. The presence of this basal disk is required for the formation of two additional structures, the medial and proximal disks. The medial disk is composed of PflA and potentially another component, FlgQ, which is required for the formation of the medial and the proximal disk [88]. This disk, associated with the inner membrane, is comprised of the protein PflB associated with the MotAB stator proteins [89]. FlgP, PflA, PflB, and PflQ have no homolog in *Salmonella*.

Each stator in *Salmonella* has two subunits, MotA and MotB. A circular multimer of the first subunit is embedded in the inner membrane and surrounds multimers of the latter, which is anchored to the peptidoglycan [90]. *Salmonella’s* flagellum is thought to contain ~11 stators organized in a circular fashion. The proximal ring of *C. jejuni* contains 17 stators [88], making a larger stator ring. In both bacteria, the rotation of the stator is powered by the dispersion of an ion motive force through the stator MotAB units [91]. The stator unit protein (MotA) is thought to be in contact with the cytoplasmic part of the motor, the C-ring [90].

In both bacteria, the rod is thought to be attached to the transmembrane MS-ring (composed of a FliF multimer) through the adaptor protein FliE [92]. FliG forms the upper rim of the C-ring, docked at the cytoplasmic side of the MS-ring. As the stator ring is larger in *C. jejuni*, it also has a wider C-ring. In *Salmonella*, the C-ring is composed of FliG, FliM, and FliN. *C. jejuni* has all three proteins with an additional protein (FliF), which is homologous to FliN. *C. jejuni* is atypical in that it contains both FliN and FliF [93]. The *C. jejuni* flagellar T3SS apparatus consists of a transmembrane export gate complex composed of FlhA, FlhB, FliP, FliQ, and FliR [94] and a cytoplasmic ATPase complex consisting of FliH, FliI, and FliJ [93]. Some of the *C. jejuni* proteins that comprise the flagellar secretion system, including FlhA, FlhB, FliP, and FliI, are also homologous to the secretion apparatus of *Salmonella*’s injectisomes (T3SS1 and T3SS2).

The polar localization and number of flagella are controlled by FlhF and FlgH [95]. FlgH is a member of the MinD family of ATPases. In non-polarly flagellated bacteria, MinD ATPases are part of the Min system, which spatially controls the division of bacteria to ensure a symmetrical division [95]. FlhF is a GTPase, and its activity of flagellar localization control seems to be stimulated by FlgH. However, the details of this control remain to be elucidated [95]. Additionally, FlhF binds to the promoter region of various flagellar genes, potentially controlling their expression [96]. A transposon mutagenesis screen identified additional genes required for motility and restricted to *Epsilonproteobacteria*, including the genes coded by the loci *Cj0062c*, *Cj0163c*, *Cj0208*, *Cj1496c* [97], although the functions of these proteins remain to be elucidated.

Overall, the flagella of *C. jejuni* and closely related bacteria possess unique features and biogenesis compared to flagella from other enteric bacteria. These features certainly contribute to the functional uniqueness of the flagellum of *C. jejuni*.

### 4.2. Proteins Exported via the C. jejuni Flagellar T3SS

The ‘classical’ T3SS possessed by many pathogenic bacteria is used to describe the delivery of effector proteins from the bacteria directly to the cytosol of a target cell [98,99,100]. The T3SS of Gram-negative bacteria are complex macromolecular structures characterized by the export of proteins across the inner and outer membranes without a periplasmic intermediate [98,101]. Whereas the signal for export is located within the 5′ end of the mRNA, as demonstrated by the secretion of hybrid fusion proteins [102], the amino-terminal end of the protein is not subjected to processing during secretion [103]. A hallmark of T3SSs that mediate interactions with animal or plant cells is that they inject ‘effector proteins’ into the cytosol of eukaryotic cells—a process termed ‘translocation’ [104]. The delivered effector proteins then modulate host cell signaling pathways to facilitate changes in cell behavior.

It was Immediately recognized that this particular secretion system shares structural similarities with the bacterial flagellum [101]. Phylogenetic studies confirmed that the proteins of the export apparatus of the classical and flagellar T3SS share a common ancestor [105,106]. Thus, flagellar biosynthesis/assembly coined the term ‘flagellar T3SS’ to describe the secretion of flagellin and allied proteins [101]. Subsequently, it was realized that non-flagellar proteins (virulence proteins) could be exported from the flagellar T3SS as well. Precedence for the secretion of a virulence factor from the flagellum was first demonstrated with *Yersinia enterocolitica* [107,108], which utilizes the flagellar T3SS to export a phospholipase termed YplA. Studies with the *Salmonella* effector proteins SptP and SopE supported the finding; removing the chaperone binding domains from SptP and SopE causes the effector proteins to be secreted through the flagellar export pathway rather than the dedicated T3SS [109].

The first evidence of *C. jejuni* secreted proteins was reported in 1999 by Konkel and colleagues [110]. The original nomenclature for the export of non-flagellar proteins was designated the *Campylobacter* invasion antigens (Cia) because they are secreted by *C. jejuni* upon incubation with host cells or host components [e.g., fetal bovine serum (FBS)] [110,111]. In addition, the deletion of one gene encoding a secreted protein, CiaB, resulted in a decrease in *C. jejuni* host cell invasion. Blast searches of the *Campylobacter* genome sequence did not reveal genes encoding a secretion system dedicated to the export of virulence proteins, such as the specialized T3SS; however, it later became clear that the flagellar system is a T3SS by definition and secretes non-flagellar proteins and even type III secreted (T3S) effector proteins under certain circumstances (see above). Subsequently, the secretion of virulence proteins by *C. jejuni* was found to be dependent on the flagellar secretion apparatus in 2004 [112]. Specifically, genetic studies using gene deletion mutants coupled with complementation analysis revealed that a complete basal body, hook, and hook-filament junction are required for the secretion of putative virulence proteins [83,112,113]. 

Since the original report/discovery that non-flagellar proteins are exported from the flagellar apparatus, several research groups have attempted to identify exported proteins. Among those, Barreto-Tobon and colleagues reported genes that are co-expressed with flagellar proteins and are dependent upon sigma 28 promoters [114]. Because of their co-expression with flagellar proteins, the group named these four proteins as flagellar co-expressed determinants (Feds). Although these proteins are co-expressed with flagella, these genes are not required for motility. Instead, they are involved in the colonization of chickens and the invasion of human intestinal cells [114]. Another research group identified the FspA proteins, which are small, acidic, non-flagellar proteins [115]. Two variants of FspA, FspA1 and FspA2, are found in *C. jejuni*. Both FspA variants are encoded by the single gene (Cj0859c) expressed by a sigma 28 promoter [115]. FspA1 and FspA2 were shown to be immunogenic in mice and to be protective against disease after challenge with a homologous *C. jejuni* strain [116]. FspA1 is also protected against illness after challenge with a heterologous strain. However, immunization with neither FspA1 nor FspA2 affected intestinal colonization. Finally, FspA2, but not FspA1, has been shown to induce apoptosis in INT 407 cells. Another protein, named FlaC, was also reported as the flagellar secreted protein and found to have a role in cell adherence and invasion [117]. In summary, different nomenclature has been used by researchers to define the proteins exported via the flagellar apparatus. Studies are needed to further verify the functional significance of these proteins and identify the molecular mechanisms(s) by which they promote *C. jejuni*-host cell interactions.

### 4.3. The C. jejuni Cia Proteins

*C. jejuni* utilizes a cell binding and effector delivery mechanism to invade host cells [118,119]. The Cia proteins (effectors) are exported from the bacterium’s flagellar T3SS and are involved in enhancing *C. jejuni* cell invasion. Cia protein secretion requires a functional flagellar export apparatus [112], and interestingly, the contact of *C. jejuni* with the host cell has been observed to occur headfirst, where the tip of the flagellum is touching the host cell [120]. Co-culture of *C. jejuni* and host cells resulted in increased expression of the genes encoding the cell-invasion proteins [121,122]. Secretion of Cia proteins is regulated by the presence of environmental factors and host cell components [111,123]. Inhibiting protein synthesis by adding chloramphenicol to the assay media inhibits the ability of *C. jejuni* isolates to invade host cells [121,124]. The current data indicate that the Cia proteins co-opt components of the focal complex (a cell-matrix adhesion structure) and the MEK/ERK signaling pathway to trigger membrane ruffling (lamellipodia and filopodia extensions—cellular protrusions supported by actin filaments) to promote the *C. jejuni* invasion of host cells. A screen of 321 *C. jejuni* genes resulted in a ranked list of candidates with a putative T3SS export signal [125]. In addition to CiaB, three additional *C. jejuni* Cia proteins have been identified to date—CiaC (*Cj1242*), CiaD (*Cj0788*), and CiaI (*Cj1450*).

The function of the first identified Cia protein, CiaB, is unknown. CiaB contains a domain of the unknown function (DUF3916) that has been annotated as a glycosyl transferase [126]. Independent research groups have reported that CiaB contributes to the *C. jejuni* invasion of host cells, but opinions differ on the contribution of this protein in cell invasion [110,127,128]. One study reported a modest reduction (28%) in the invasiveness of the *C. jejuni ciaB* mutant (strain 81-176), but this study was performed with an isolate harboring a truncated form of the gene (the gene disrupted at base pair 1227 of the 1833-bp open reading frame) rather than a gene-deletion mutant [128]. CiaB was identified to have a role in the secretion process, and a CiaB-deficient isolate does not export effector proteins [110]. Whereas CiaB may manipulate the secretion apparatus, additional studies are required to test this possibility. Another group identified CiaB’s role in *C. jejuni* internalization into host cells and colonization in chickens [129].

CiaI (*ciaI*, *Cj1450*) was identified using a screen for *C. jejuni* flagellar T3S proteins in *Y. enterocolitica* [125]. A deletion of *ciaI* in *C. jejuni* revealed the absence of a 21 kDa band in the supernatant of the mutant bacteria but not in the wild-type strain [130]. Expression of a CiaI-FLAG protein led to the detection of a FLAG-reactive band in the supernatant [130]. The cytoplasmic protein CysM was not detected in the supernatant, demonstrating that the FLAG-reactive band in the supernatant was not caused by cell lysis. The CiaI-FLAG protein was not detected in the supernatant of the *flgBC* flagellar basal body mutant containing the pRY111-CiaI-FLAG vector, providing genetic evidence that CiaI is secreted from the bacterial flagellum. Notably, the Cia proteins—including CiaI—are delivered to the cytosol of host cells as demonstrated by fusing the adenylate cyclase domain (ACD) of the *Bordetella pertussis* CyaA protein to the C-terminus of the Cia proteins and measuring cytoplasmic cAMP levels after infection [83], an approach that has been utilized by others to establish bacterial effector delivery [131,132,133,134,135]. Researchers have examined the ability of the *ciaI* mutant to bind, invade, and replicate within INT 407 and T84 cells using an in vitro replication assay (the gentamicin protection assay) and confocal microscopy. Intracellular replication was impaired in the *ciaI* mutant, but not cell binding or cell internalization, and a greater number of *ciaI* mutant bacteria were contained in *Campylobacter*-containing vacuoles (CCVs) associated with Cathepsin D, a lysosomal aspartyl protease that indicates increased fusion with the lysosome when compared to the wild-type strain [130]. Ectopic expression of CiaI fused to GFP or mCherry in HeLa cells revealed a punctate cellular pattern that was consistent with vesicular structures [130]. CiaI contains a stretch of amino acids from position 149 to 154 (DSKKLL) that resembles a classical di-leucine motif, [DE]xxxL[LI] [136,137], which is reportedly involved in endocytic pathway targeting. Mutation or deletion of the di-leucine motif results in a diffuse-fluorescence phenotype [130]. Collectively, these data suggest that CiaI contributes to the survival of *C. jejuni* within cultured cells, perhaps by inhibiting the fusion of the CCV with lysosomes. Additional studies are required to determine the precise function of CiaI and if it modifies the survival of *C. jejuni* within cells by binding to a host cell protein.

CiaC (*Cj1242*) and CiaD (*Cj0788*) are the two Cia proteins that have been studied extensively. Both CiaC and CiaD proteins have been demonstrated to be exported from the flagellar apparatus using a wild-type isolate and flagellar mutants transformed with a vector containing the CiaC-ACD and CiaD-ACD tagged proteins, respectively, and delivered to host cells by the ACD assay [83,113]. A *C. jejuni ciaC* deletion mutant shows a reduction in *C. jejuni* invasion of intestinal cells [83,125]. The necessity for CiaD in host cell invasion has been demonstrated by two independent research groups, one using a gene deletion [113] and the other using a large-scale screen [138]. The contribution of CiaD in disease has further been demonstrated in a mouse model [113] and a piglet model [139]. A *C. jejuni ciaD* mutant resulted in a decreased inflammatory response in the porcine-ligated loop model [139]. The putative host cell binding target of CiaD was initially identified as IQGAP1 (a scaffold protein that coordinates actin reorganization) and then confirmed by in vitro cellular infection using a targeted pull-down assay coupled with immunoblot analysis. Finally, CiaD was found to bind directly to the host cell protein IQGAP1, thus displacing RacGAP1 from the IQGAP1 complex [119]. This, in turn, leads to the unconstrained activity of the small GTPase Rac1, which is known to have roles in host cell-actin reorganization and the internalization of *C. jejuni*. CiaD activates the Mitogen-activated protein (MAP) kinase pathways Erk 1/2 and p38 and helps maximal invasion of *C. jejuni* to host cells and release of IL-8 pro-inflammatory cytokine [113]. CiaD has also been shown to be required for the maximal phosphorylation of cortactin, an actin-binding protein in serine residue at positions 405 and 418 [140]. Taken together, the CiaD protein has been demonstrated to (i) be secreted from *C. jejuni* via the flagellum, (ii) be delivered to the host cytosol of a target cell, (iii) bind to a host cell protein whereby it alters host cell signaling, and (iv) contribute to disease in two different animal models. 

In summary, whereas *C. jejuni* does not have a dedicated or specialized T3SS, this bacterium has a device for the secretion of proteins that modify host cell signaling events in a manner that is advantageous for disease, similar to effectors delivered by other more intensely studied pathogenic bacteria, including *Yersinia*, *Shigella*, and *Salmonella*.

### 4.4. Predictions of Proteins Exported from the C. jejuni Flagellar T3SS

Several proteins are known to be exported by the *C. jejuni* flagellar T3SS, and their function has been demonstrated in cellular and virulence activities (as described above). However, other proteins may be secreted by the T3SS that could have importance in *C. jejuni* virulence. We applied the SIEVE [SVM (Support Vector Machine)-based Identification and Evaluation of Virulence Effector] algorithm to protein sequences of three *C. jejuni* genomes (NCTC 11168, 81-176 and F38011) to make predictions of T3S effectors. SIEVE uses machine learning applied to the N-terminal 30 amino acids of proteins to predict their ability to be secreted by the T3SS in a highly accurate manner [141]. SIEVE represents the N-terminal amino acids in one-hot, where each position is represented by a vector of 20 binary values (one for each different amino acid). The vector of all 30 amino acids, as well as a vector of the amino acid composition for the protein, and sequence similarities to other organisms are used as input to the trained SIEVE model [141]. SIEVE was independently evaluated as an overall performance prediction tool. Like similar tools, it occasionally fails to predict certain proteins known to be secreted by these systems [142]. The rank of predicted proteins is determined by the SIEVE scores that use the output scores by Z-score transformation. 

**Figure 4:**
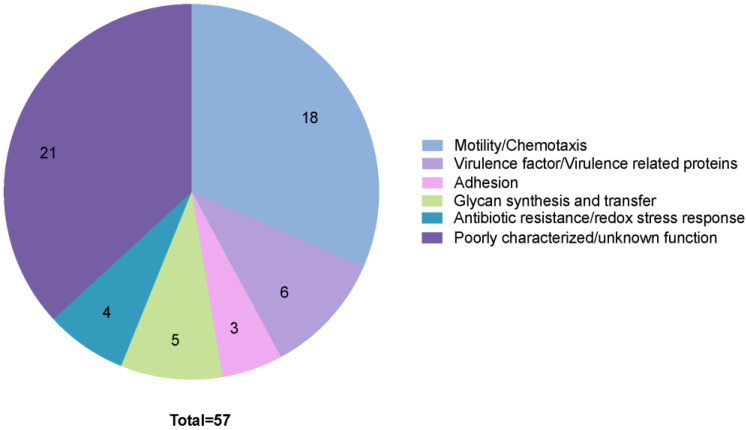
Pie chart of functional categories of *C. jejuni* flagellar type III secreted proteins predicted by the SIEVE algorithm. Only proteins with SIEVE scores of above 1.3 are considered for these categories. About 31.6% (*n* = 18) of secreted proteins have predicted function in cell motility or chemotaxis, 10.5% (*n* = 6) are virulence-related proteins, 5.3% (*n* = 3) have a role in adhesion, 8.8% (*n* = 5) have a role in glycan synthesis and transfer, and 7% (*n* = 4) have a role in antibiotic resistance or stress response. About 36.8% (*n* = 21) of proteins do not have any assigned function or are poorly characterized.

The three *C. jejuni* strains 81-176, F38011, and NCTC 11168 were chosen for the prediction analysis based on the common laboratory usage of these strains for pathogenicity studies. Blast searches were used to identify proteins that were homologous between the different strains. To make Table S2 readable and easy to navigate, we included the homologs of the three strains in the final table, and in all cases, their prediction score was above 1.3. We considered proteins not passing the screening in one strain but predicted in another strain as secreted. The SIEVE algorithm predicted a total of 57 proteins to be secreted by *C. jejuni* T3SS (Table S2). In total, 87.7% of the exported proteins were shared between the three strains. *C. jejuni* NCTC 11168 was the strain that had the most proteins predicted to be secreted by a T3SS with 57 proteins, 52 were predicted for strain 81-176, and 50 for strain F38011. Some of the proteins identified are known to target and modulate host cell signaling pathways where they redirect cellular processes. Two pie charts were generated to illustrate the functional categories of the predicted *C. jejuni* T3S proteins from strain *C. jejuni* NCTC 11168. The first pie chart groups proteins into categories based on similarity with homologous proteins (Figure 4), and the second chart groups proteins using Clusters of Orthologous Genes (COG) functional categories (Table S2). Among the 57 predicted proteins secreted by *C. jejuni* T3SS, 34 proteins have defined COG (Clusters of Orthologous Genes) categories, 17 proteins have predicted functions in the newer EggNog database, and six proteins do not have any assigned COG categories (Table S2, Figure S1). Here we discuss the proteins belonging to the functional categories based on homologies.

In most bacteria, the flagellar T3SS is known for its secretion of proteins belonging to the flagellar apparatus. As expected, the largest functional group of proteins predicted to be secreted was related to the flagellum and mobility, with a total of 18 proteins with paralogs in the three strains (Figure 4). A motility-regulating protein, Cj0170, was also predicted to be secreted by T3SS but is only found in the genome of the strain NCTC 11168. Six T3S proteins were predicted to be associated with virulence, including three Cia proteins: CiaC, CiaD, and CiaI [83,113,119,125,130,139,140]. Whereas the secretion of CiaB by the flagellar apparatus is well documented, the prediction tool failed to predict its secretion, indicating that other proteins secreted by the flagellar apparatus may have been missed by this screen. Other proteins that are involved in virulence include TylA [143,144], Cj1631c [145], and Cj1004 [146]. All of these are coded by genes present in the three genomes included in the analysis. Other proteins proposed to contribute to virulence include TylA [143,144], Cj1631c [145], and Cj1004 [146]. In addition, JlpA [147] and PglH [148] are proposed to be involved in host-cell interactions. All of these proteins are coded by genes present in the three genomes included in the analysis.

Multiple glycan synthesis and transferase enzymes were predicted to be secreted. Interestingly, while all five glycan-related proteins were found in *C. jejuni* strain NCTC 11168, only two were coded in the genome of strain 81-176 and only one in the genome of strain F38011. This latter gene codes for PseI, which is involved in the synthesis of pseudaminic acid, a component of glycoproteins and glycolipids on the cell surface of the pathogen and is required for its pathogenesis [149]. The proteins encoded in a limited number of genomes may be dispensable or required for the colonization of only certain hosts. Two proteins involved in antibiotic resistance (CmeC and Cj1086c) and two proteins potentially involved in response to oxidative stress (ThyX and Cj1623) were predicted to be secreted in all three strains.

Finally, the functions of 21 proteins are unknown, but it is possible that these may participate in functions related to motility, adhesion, virulence, antibiotic, or redox stress resistance. Knowing the importance of these processes in pathogenicity, we propose to name these the “proteins putatively secreted by the flagellum (PPSFs).” A verification of their secretion by the flagellar apparatus and their role in the processes mentioned above remains to be explored.

## 5. Type IV Secretion System in *C. jejuni*

Type IV secretion systems (T4SS) are a highly diverse set of secretion systems present in both Gram-negative and Gram-positive bacterial species. T4SS are critical virulence factors for many different pathogens, including *H. pylori*, *Legionella pneumophila*, and *Coxiella burnetti* [150], and typically function as a contact-dependent method of transporting proteins or DNA-protein complexes across the bacterial cell envelope. Similar to T3SS, it utilizes a needle-like apparatus to directly transport effector molecules from bacterial pathogens into eukaryotic host cells. T4SS are typically encoded on either conjugative plasmids, integrative and conjugative elements (ICEs), or genomic pathogenicity islands (PAIs) [151,152], which allows them to be easily spread via horizontal gene transfer among different species, including archaea. Broadly, T4SS can be grouped into three functional types: conjunction, translocation, and protein transfer [153]. The T4SSs in Gram-negative bacteria range from 12–27 proteins that compose the structure and the overall composition [114,154,155,156,157] but are typically composed of 12 conserved subunits that are required for a fully functional system [158,159].

To date, the best characterized Gram-negative T4SS is the *Agrobacterium tumefaciens* VirB/VirD4 T4SS, which is composed of 12 genes from the *virB* and *virD* operons and consists of four distinct components: (i) substrate receptor coupling protein (CP) VirD4, (ii) an inner membrane complex (IMC), (iii) an outer membrane complex (OMC), and (iv) the conjunctive pilus [158,159,160,161]. The first three components form the substrate translocation channel, whereas the pilus forms the extracellular unit. The IMC is composed of three ATPases (VirD4, VirB4, VirB11), along with the membrane protein VirB8 and inner membrane proteins VirB3 and VirB6. Structures within the OMC are called the core complex and translocation channel. The core complex is composed of two lipoproteins VirB7 and VirB9, and the multifunctional membrane/scaffolding protein VirB10. The translocation channel is composed of the membrane protein VirB8, lipoprotein VirB9, and scaffolding protein VirB10. The major pilin subunit VirB2 along with the minor subunit VirB5 comprise the extracellular pilus [162]. The transglycosylase VirB1 is not essential for the assembly of the translocation channel but is required for pilus formation. Studies have shown that the *A. tumefaciens* VirB/VirD4 T4SS structure is conserved among Gram-negative T4SS in other bacterial species, including *H. pylori.* One of the most well-characterized T4SS is the *H. pylori* cytotoxin-associated gene (*cag*) pathogenicity island (*cag* PAI), which is composed of 32 genes that encode for the Cag-T4SS, including several VirB/VirD4 homologous subunits [163]. The Cag-T4SS is a critical virulence factor of *H. pylori* that has been associated with more severe disease production [163]. *H. pylori* is a member of the order *Campylobacterales* and related to *Campylobacter*, but unlike *H. pylori*, the presence of a T4SS in *Campylobacter* is not straightforward. 

### 5.1. Plasmids Associated with C. jejuni T4SS Components

**Figure 5:**
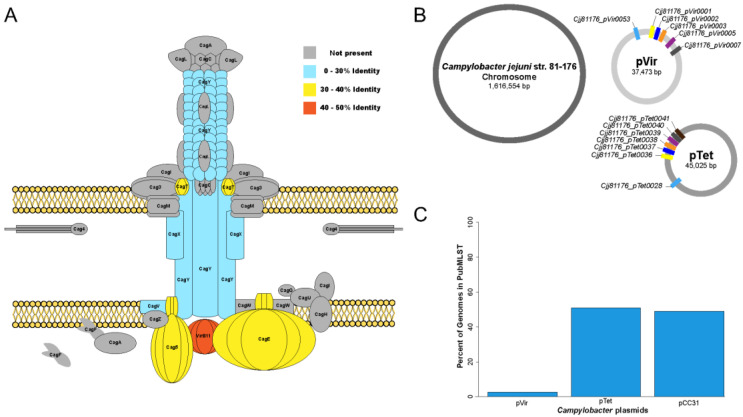
Components of the type IV secretion system (T4SS) identified in *C. jejuni***.** Panels: (**A**) Identification of *C. jejuni* homologs to the structural components of the *H. pylori* Cag T4SS. Colors represent the percentage of protein identity for those proteins present as indicated in the figure legend. (**B**) Identification of potential T4SS genes in *C. jejuni* str. 81-176 genome that is represented by the chromosome, pVir and pTet; identical colors on pVir and pTet represent T4SS genes that encode for the same T4SS structural component, whereas different colors encode for T4SS structural genes unique to that particular plasmid. (**C**) Percentage of the >54,000 genomes in the PubMLST database that contain at least 2000 bp of the *Campylobacter* plasmid (pVir, pTet, or pCC31) that potentially encodes T4SS structural components.

In 2000, Bacon et al. were the first to describe a potential T4SS in *C. jejuni*, based on the finding that the *C. jejuni* pVir plasmid has seven genes predicted to be orthologs of T4SS proteins found in *H. pylori* [35] (Figure 5A,B). Further studies found that knocking out these genes reduced the ability of *C. jejuni* strain 81-176 to invade human epithelial cells *in vitro* and decreased the strain’s overall virulence in the ferret diarrhea model [35,164]. However, many of these studies were conducted before or around the release of the first *C. jejuni* genome sequence [165], but the first sequenced genome was from *C. jejuni* strain NCTC 11168, which does not carry the pVir plasmid. In fact, the pVir plasmid is only carried by a small subset of *C. jejuni* isolates (up to 3% of the 54,065 *C. jejuni* and *C. coli* genomes present in the PubMLST database based on at least 2000 bp BLAST hit) (Figure 5C), which suggests a limited or nonessential role for the pVir plasmid and the T4SS in *Campylobacter* virulence. Analysis of two tetracycline-resistant plasmids, pTet and pCC31, from *C. jejuni* strain 81-176 and *C. coli* strain CC31, respectively, found proteins with homology to a T4SS in *Actinobacillus actinomycetemcomitans* encoded on the pVT745 plasmid [166]. The authors concluded that there were 10 predicted proteins distinct from the T4SS found on pVir to be involved in both conjugative transfer and plasmid replication. Homologs to VirD4, VirB2, VirB4, VirB5, VirB6, VirB7, VirB8, VirB9, and VirB10, and VirB11 from *A. actinomycetemcomitans* were found to be encoded on both plasmids; however, the functionality of these proteins in *Campylobacter* is not certain. For example, VirB2 is essential for pilus construction, but electron microscopy of both *C. jejuni* strain 81-176 and *C. coli* strain CC31 did not provide evidence of a pilus [166].

Unlike pVir, both the pCC31 (up to 49% of PubMLST genomes) and pTet (up to 51% PubMLST genomes) plasmids appear to be more commonly found in *Campylobacter* isolates (Figure 5C). Yet, pTet has been shown to have a significant variation in size, ranging from approximately 36,975 bp to 125,531 bp [167]. Examination of the encoding genes of the smaller versions of the plasmid demonstrates a lack of some or all the potential T4SS encoding genes. Furthermore, no studies have demonstrated that any of these plasmids encode for a completely functional T4SS for any species of *Campylobacter*.

In addition to the identification of potential T4SS encoding genes on various *Campylobacter* plasmids, *C. jejuni* has been shown to encode homologs of a few components from the T4SS of other pathogens. Prior to whole genome sequencing (WGS), *C. jejuni* strain ATCC 43431 was demonstrated through competitive hybridization with *C. jejuni* strain NCTC 11168 to contain orthologs to a T4SS in *Helicobacter hepaticus*, which has homology to the Impaired in nitrogen fixation (Imp) operon in *Rhizobium leguminosarum* [168]. The *imp* locus in *R. leguminosarum* is proposed to be involved in temperature-dependent protein secretion [168] and contains a homolog of a T4SS inner membrane protein, IcmF [169]. IcmF is an essential part of the Dot/Icm T4SS and functions as a stabilizer for the inner membrane secretion complex [169]; however, the Dot/Icm T4SS is not found in the *Campylobacter* species. In addition, the function of IcmF in a *Campylobacter* T4SS has only been proposed and is not supported by experimental evidence. Homology to another T4SS component, TraG from *Neisseria gonorrhoeae*, is required for DNA transfer during bacterial conjugation [168], and has also been identified in *Campylobacter*. Although TraG, IcmF, Imp-like proteins, and the various genes on pVir, pTet, and/or pCC31 have proteins that share homology with T4SSs in other pathogens, including the closely related *Helicobacter*, there is no evidence indicating that *Campylobacter* contains all the components for a functional T4SS. In fact, to date, no functional T4SS has not been identified, suggesting that *Campylobacter* does not produce a functional T4SS.

### 5.2. Homologs of T4SS Proteins in C. jejuni

To further explore the potential of a *Campylobacter* T4SS, each of the 32 genes of the *H. pylori* Cag-T4SS were screened against *C. jejuni* in the NCBI non-redundant (nr) database to identify the overall protein homology and identify missing components (Table S3). Overall, only 7/32 (21.9%) genes were identified in *C. jejuni*, including the ATPases (CagE, Cag5, and VirB11) and some of the structural genes (CagV, CagT, CagX, and CagY), but the majority of the structural genes are missing (Figure 5A). Furthermore, to understand the *Campylobacter* population genomics for a potential T4SS, the 54,065 *C. jejuni* and *C. coli* genomes present in the PubMLST database were screened for protein homologs of the *H. pylori* Cag-T4SS. The analysis revealed only seven genes associated with T4SS in all *C. jejuni* isolates (Table S3). Interestingly, the location of six and seven of these genes were found to be present on both the pVir and pTet plasmid of *C. jejuni* strain 81-176, respectively (Figure 5B), suggesting that the frequency of the genes in other isolates might be due to the presence/absence of these plasmids. Ultimately, these results demonstrate that many T4SS components are missing in the *Campylobacter* pangenome. This finding is consistent across the global *Campylobacter* population, thus suggesting that *Campylobacter* does not produce a T4SS and that these homologs in the genome are involved in other functions.

## 6. Type VI Secretion System in *C. jejuni*

Type VI secretion system (T6SS) is another contact-dependent secretion system present in at least 25% of Gram-negative bacterial species [170]. First identified in *Vibrio cholerae*, *R. leguminosarum*, *Pseudomonas aeruginosa*, and *L. pneumophila*, T6SSs deliver effector proteins to both prokaryotic and eukaryotic cells using a contractile needle [171,172,173,174]. Investigations have focused on the various roles that T6SSs can play in bacterial pathogenesis, including contact-dependent cytotoxicity towards red blood cells, actin cross-linking, pore-forming activities, and even anti-fungal activities [171,175,176,177]. Survival against bile salts and/or reactive oxygen species are also roles that have been connected to T6SSs in bacteria [178,179,180,181]. The archetypal T6SS structure contains at least 13 core components (TssA-TssM) and forms a structure similar to the contractile T4 bacteriophage tail [182]. The components form four distinct structures: membrane core complex, baseplate, cytoplasmic contractile sheath, and a puncturing needle [183,184,185,186,187]. The membrane complex is composed of the lipoproteins TssJ, TssL, and TssM. TssL is located on the cell envelope, providing an anchor and docking station for the T6SS [184,188]. The inner membrane proteins are composed of the proteins TssM and TssL, which interact to result in oligomerization [189,190]. Lytic transglucosylase (LTG) is recruited by TssM to degrade local peptidoglycan layers, allowing for the assembly of the TssJLM complex [191,192]. Baseplate assembly also takes place on the membrane complex [187]. The baseplate is composed of six proteins, including TssA, TssE, TssF, TssG, TssK, and a valine-glycine repeat G protein (VgrG or TssI), which serves as a platform for the cytoplasmic contractile sheath and construction of the inner tube. The inner tube is made of hemolysin-coregulated protein (Hcp or TssD) [187,193]. Hcp is vital for a functional T6SS and is often used in the screening of isolates for the presence of T6SS as well as in mutagenesis studies [194,195,196]. The binding of TssA to the membrane complex initiates tail polymerization, coordinating the biogenesis of the tail tube and sheath [197]. TssA may also be responsible for the recruitment of TssE, TssK, and VgrG that function to position and anchor the baseplate complex to the sheath [197,198]. Once the baseplate component is assembled, stacks of Hcp hexameric rings work to elongate the contractile needle in conjunction with the final sheath that wraps around the needle composed of TssB and TssC subunits [199]. Once fully assembled, the sheath can contract and eject an Hcp-VgrG spike and effectors in any direction [200].

A hallmark of a functional T6SS is Hcp and VgrG secretion into the extracellular environment [172,196,201]. Hcp has multiple roles during and post secretion. During secretion, the hexametric ring structure allows for the effectors to pass through the center [196]. Once secretion occurs, Hcp prevents effector degradation and has its own secretory functions that contribute to the bacterial competition [201,202,203,204,205,206]. Once the Hcp-VgrG spike is ejected, ATPase ClpV (TssH) works to break down the components and recycle them for future use [207,208]. 

### 6.1. Prevalence of T6SS-Associated Genes in C. jejuni

A T6SS in a *Campylobacter* species was first reported in *C. jejuni* by Lertpiriyapong et al., and through mutational studies, the authors found the T6SS could have a role in bile salt survival, host cell adherence, and invasion [180]. T6SSs have been identified in 11 other species of *Campylobacter*, including *C. coli*, *C. concisus*, *C. ureolyticus*, *C. amoricus*, *C. cuniculorum*, *C. helveticus*, *C. ornithocola*, *C. peloridis*, *C. rectus*, *C. subantarcticus*, and *C. lari* [175,209,210]. One study found that when using the *hcp* gene as an indicator of a T6SS, the prevalence of positive *C. coli* isolates (56.1%) was significantly higher than for *C. jejuni* (28.8%) [210]. Nevertheless, the prevalence of the T6SS is higher in *C. jejuni* clinical isolates compared to the animal/environmental isolates as several studies have found that when using the *hcp* gene as an indicator of a T6SS, only 3–33% of animal isolates compared to 27–74% of clinical isolates could potentially encode for the T6SS [211,212,213,214]. Interestingly, one study in Pakistan found none of the *C. jejuni* clinical isolates tested positive for a T6SS, and 4.6% of animal/environmental samples tested positive [215]. Another study reported the prevalence of T6SS was in 72% of *C. jejuni* isolates recovered from wild birds [216]. However, the wide range among isolates has been suggested to reflect variance in sample size and detection methods between studies [214]. Additionally, the use of the *hcp* gene as a marker for complete T6SS loci may need to be revised, as one study found that the mere presence of this gene does not correlate with the presence of all T6SS open reading frames (ORFs) [214]. A recent genomic analysis of 371 *Campylobacter* genomes, using the T6SS-positive *C. jejuni* strain 488 as a reference, found that 33.3% contained a T6SS operon [217]. We examined the 54,065 *C. jejuni* and *C. coli* genomes in the PubMLST database for the Hcp protein. We found it was present in 24.8% of the genomes with >75% identity across >75% of the protein. However, more detailed analysis is needed to truly understand the percentage of *Campylobacter* isolates that encode a functional T6SS. Recently, a comprehensive protocol for specific isolation and functional characterization of T6SS-positive *C. jejuni* was published by Gupta et al. [218]. This protocol also addresses how *C. jejuni* T6SS may be involved in targeting other bacterial populations [218]. Although some of the questions surrounding *Campylobacter* T6SS have begun to be addressed and will be discussed in this review, there are many additional research opportunities that need to be investigated.

### 6.2. Genetics of C. jejuni T6SS

**Figure 6:**
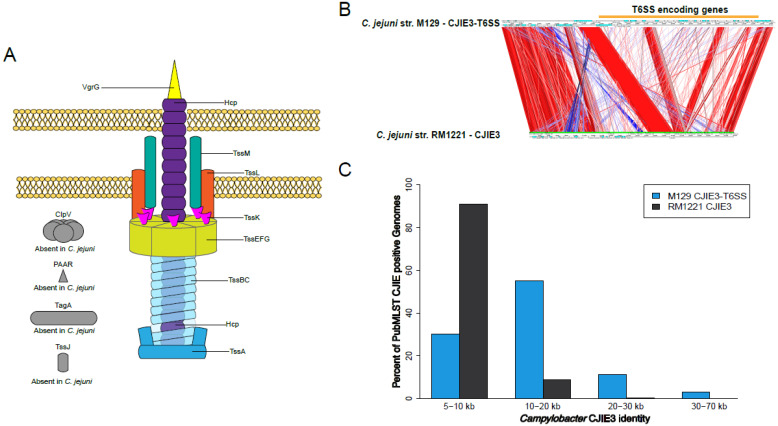
Components of the type VI secretion system (T6SS) identified in *C. jejuni*. Panels: (**A**) Identification of *C. jejuni* homologs to the structural components of Gram-negative T6SS; all components are present except for ClpV, PAAR (tip of the T6SS needle), TagA (functions to stop assembly of the tail), and TssJ (membrane complex) [an alternative model has been published by Cherrak et al. [208]]. (**B**) Alignment comparison of CJIE3 from *C. jejuni* strain RM1221 to *C. jejuni* strain M129 that contains the T6SS; red demonstrates regions present in M129 CJIE3 that are also present in RM1221 CJIE3 and vice versa for blue. The region containing T6SS encoding genes is marked above the M129 CJIE3 region. (**C**) Percentage of the approximately 13,000 *Campylobacter* genomes in the PubMLST database that contain CJIE3 based on the size of the hit identity for the element for the two different CJIE3s with or without the T6SS genes.

The T6SSs are predicted to be organized into three genetic groups, and two of the groups can be further categorized into additional subgroups [217]. T6SS I-a and I-d subgroups have nine core components that differ by their location of the *vgrG* gene and the *tagH-tssM-hcp* genetic cluster. T6SS II and III groups differ from each other and the T6SS I group because of the encoding direction, location of several structural genes, and variation between the *hcp* and *vgrG* genes [217]. Furthermore, the T6SS I and II groups do not cluster based on *Campylobacter* species, and to date, the T6SS III group has only been found in *C. rectus* [217]. A comprehensive bioinformatics analysis of the *C. jejuni* T6SS in 513 publicly available *Campylobacter* genomes suggested a sequential integration of the T6SS structural and effector genes rather than a single integration event [217,219]. The *C. jejuni* T6SS cluster is encoded on a pathogenicity island (CJPAI-1), also known as the *C. jejuni* integrated element 3 (CJIE3) [219]. However, although many species of *Campylobacter* may contain CJIE3, not all may encode for a T6SS. For example, the CJIE3 of *C. jejuni* strain M129 contains the majority of the structural components of the T6SS, as outlined in Figure 6A, but in comparison to the CJIE3 of *C. jejuni* strain RM1221, there are significant genomic differences predominately caused by the presence/absence of the T6SS (Figure 6B). To further understand the presence of CJIE3 with or without the T6SS in *Campylobacter* genomes, we again examined the 54,065 *C. jejuni* and *C. coli* genomes in the PubMLST database for either the RM1221 CJIE3 or M129 CJIE3-T6SS. The BLAST search found 23.4% of the genomes had at least 5000 bp of the RM1221 CJIE3 and 28.5% had that much of the M129 CJIE3-T6SS, but there were variations in the amount of the different CJIE that hit (Figure 6C). We did not separate the location of the identity hit to differentiate between the presence or absence of a T6SS in the CJIE3 of the genomes, but 23.4–28.5% of the genomes contain at least 10% of the RM1221 CJIE3 or M129 CJIE3-T6SS, respectively. Furthermore, isolates of *C. jejuni* have also been found to harbor the T6SS on megaplasmids [219,220]. Recently, megaplasmids in *C. jejuni* have been suggested to have a role in virulence as they will contain antibiotic-resistant genes and secretion system genes. Marasani et al. reported two megaplasmids that harbor genes for both T4SS and T6SS [220]. When these plasmids were transferred via conjugation to a plasmid-less *C. jejuni* isolate, the recipient bacteria demonstrated increased cytotoxicity towards red blood cells, a proposed function of *C. jejuni* T6SS, as discussed below.

### 6.3. Structure and Effectors of the C. jejuni T6SS

Through bioinformatic analysis, *Campylobacter* T6SS is predicted to be structurally similar to those in other bacterial species as it contains a majority of the core T6SS genes. Table S4 shows the *C. jejuni* genes encoding T6SS components compared to *V. cholerae*; the gene encoding TssJ is apparently missing. In addition, the specific structure of the *C. jejuni* T6SS compared to other bacteria is currently unknown [219]. The gene organization is most similar to the T6SS found in *H. hepaticus*. Interestingly, the *C. jejuni* T6SS lacks the ATPase ClpV (Figure 6A), similar to *V. cholerae* [221], thus, an alternative unidentified energy source must be necessary for recycling the TssB/TssC sheath [175]. Most of the studies for T6SS in *Campylobacter* have focused on the Hcp protein. In fact, there are numerous detailed studies characterizing the structure of the Hcp protein from different *Campylobacter* species. Protein structure analysis of the *C. jejuni* Hcp (HcpCJ) has found both structural and sequence differences from the six known Hcp crystal structures of other bacteria available in the protein data bank [222]. Typical T6SSs from other bacteria have been found to contain multiple Hcp proteins; however, *C. jejuni* has been found to only contain one Hcp protein with multiple functions. Studies in *C. jejuni* have shown that the Hcp protein is involved in biofilm production, attachment and invasion, and cytotoxic effects. These phenotypes have also been linked to other T6SS effector proteins [175,180,212,222]. Robinson et al. predicted that *C. jejuni* strain 488 has four T6SS effectors (*CJ488_0980*, *CJ488_0982*, *CJ488_0988*, and *CJ488_0994*) that function as nucleases and as NAD^+^-glycohydrolase [219]. However, these effectors have not been found in all T6SS-positive *C. jejuni* isolates, suggesting that there are unique or strain-specific effectors that still need to be identified. Whereas the T6SS is apparently present in multiple *Campylobacter* species, complete structural characterization of all the components of the *Campylobacter* T6SS remains to be elucidated.

### 6.4. The Function of the C. jejuni T6SS

The *C. jejuni* T6SS has been demonstrated to have diverse functionality within pathogenicity and adaptation to different environments [223]. Although it is not essential for bacterial survival or growth, it is suggested that *C. jejuni* isolates with a functional T6SS are associated with more severe clinical manifestations [213], including a higher association with immunocompromised individuals [213,224]. The T6SS role in the colonization of the chicken reservoir has also been suggested, as one study found that T6SS-positive *C. jejuni* isolates recovered from both chickens and humans colonized chickens at a higher rate and had an increased invasive ability for chicken primary intestinal cells [212]. Similarly, another study using a murine model found that a T6SS-deficient isolate had a reduced ability to colonize mice [180]. The *C. jejuni* T6SS has also been shown to have contact-dependent cytotoxicity towards the erythrocytes [175]. However, in other studies, T6SS-negative isolates also induced erythrocyte lysis [211]. T6SS has also been found to improve pathogen survival in bile salts and their metabolites (deoxycholic acid), and Liaw et al. found that the T6SS has a role in oxidative stress response through the expression of oxidative stress response genes such as *katA*, *sodB*, and *ahpC* [212]. Another study found that when co-culturing a *C. jejuni* T6SS-positive isolate with *Escherichia coli* DH5α, the density of *E. coli* was significantly reduced compared to co-culturing with a T6SS-negative isolate and morphological changes were observed [225]. In a separate study, T6SS-positive *C. jejuni* isolates were able to grow in co-cultures with *Helicobacter pullorum*, whereas T6SS-negative *C. jejuni* were completely inhibited [211]. Both of these studies suggest potential T6SS-mediated predation. This interbacterial effect was not seen with T6SS-positive *H. hepaticus* when co-cultured with *C. jejuni* strain NCTC 11168 (a T6SS-negative strain), suggesting that this is a function unique to the *Campylobacter* T6SS [226]. Furthermore, because of the potential role of T6SS in enhancing *C. jejuni* virulence, one study developed the Hcp protein as a vaccine to reduce the colonization of *C. jejuni* in chickens and had significant results with their mucosal immunizations [227]. When a recombinant Hcp was administered via an intra-gastric route, the cecal load of *C. jejuni* was significantly reduced compared to control groups, demonstrating the possibility for Hcp immunization in chickens [227]. 

Whereas the T6SS is present in some *Campylobacter* clinical isolates, particularly *C. jejuni*, it is not required for pathogenesis and virulence. Yet, the presence of the T6SS might provide an evolutionary advantage in the environment, which could also result in a more severe clinical manifestation, but this has yet to be proven.

## 7. Summary—Conclusions

*Campylobacter* bacteria are the most common bacterial cause of foodborne illness in the United States and a significant health burden worldwide [228]. Moreover, the incidence of *Campylobacter* infections continues to increase and is likely even greater than expected due to underreporting. Although much has been learned about the virulence attributes and pathogenic mechanisms in the past few decades, the significance of this pathogen remains underappreciated by most of the population. This lack of recognition is partly caused by most *Campylobacter* illnesses occurring sporadically and not associated with large outbreaks. In addition, compared to some intensely studied microorganisms, *Campylobacter* bacteria are more difficult to work with in the lab and to genetically manipulate. The CDC and the World Health Organization (WHO) recently recognized *Campylobacter* bacteria as a priority organism due to antibiotic resistance [229,230]. Notable is that fluoroquinolone (FQ)-resistant *Campylobacter* organisms have persisted and even increased in recent years [231,232]. Moreover, it appears that a single point mutation in *gyrA* (i.e., Thr-86-Ile) confers enhanced fitness and virulence [233,234,235,236,237]. A recent report suggests that because of climate change in Northern Europe, the cases of campylobacteriosis could double by the end of the 2080s [238].

The most significant change that has occurred over the past decade is the use of WGS to compare *Campylobacter* isolates from various sources. These genomic sequences have allowed researchers to identify the core genome and variable genes and to focus on gene function rather than debating whether certain genes are present or absent within the species. The vast number of sequences has also allowed researchers to draw evidence-based conclusions regarding the presence and absence of secretion systems and virulence traits. Based on our review of the literature and the analysis of the *Campylobacter* genomes in the databases, the currently available data indicates that *C. jejuni* possesses T3SS and T6SS but not a T4SS. Whereas the flagellar T3SS is conserved in *C. jejuni* isolates, genes of unknown function are present in isolates that could influence the functionality of the system. We found that the T6SS is frequently contained within an integrated element that is strain variable. The genomic content indicates that *C. jejuni* has evolved in a manner distinct from many other Gram-negative bacteria, thereby preventing generalized conclusions regarding pathogenic mechanisms and virulence determinants. Nevertheless, it is probable that other Gram-negative bacteria, including less intensely studied bacteria, may share virulence mechanisms, including the ability to export proteins from the flagellum.

Although not the focus of this article, we would be remiss if we did not re-emphasize that the incidence of disease and percentage of antibiotic-resistant isolates continues to increase. These two factors are intertwined and, in conjunction with other types of mutations that occur due to *Campylobacter* growth in varied and challenging (stress) conditions, further drive bacterial fitness and pathogenesis. Any variation in the pathogen, host, or environment may provide conditions for the pathogen to acquire point mutation that alter disease severity [233,234,235,236,237]. Although not necessarily surprising, the unique aspects of the *Campylobacter* flagellum set this organism apart from others and, as demonstrated by multiple laboratories, facilitate *C. jejuni*-cell invasion. The T6SS promotes colonization and contributes to oxidative stress resistance. We hypothesize that *C. jejuni* relies on the T3SS and, in certain instances, on the T6SS, to establish a niche and to adapt to new environments, thereby providing for continued success in animal reservoirs and humans (the accidental host).

In summary, based on a review of the literature and analysis of the genomic content of *Campylobacter* sequences deposited in the databases, we conclude that *C. jejuni* possesses the T3SS and T6SS that export proteins that contribute to disease progression in a susceptible host. There are still many outstanding questions remain that need to be addressed to understand the *C. jejuni* virulence and disease mechanism. We listed some of the outstanding questions for future study in Box 1. Overall, a better understanding of gene function is needed to establish suitable targets for the development of diagnostic tests to identify isolates with different disease potentials or animal reservoirs based on genomic content.

Box 1 **Outstanding questions.**

- What are the other effector proteins secreted by the *C. jejuni* flagellar T3SS that have not been identified?
- Are there any changes in the structure or composition of the *C. jejuni* flagellar T3SS during secretion?
- Is there a homolog of T6SS ClpV (ATPase) in *C. jejuni*?
- What are the roles of effector proteins in *C. jejuni* virulence (colonization, adhesion, invasion, the establishment of the *Campylobacter*-containing vacuole, intracellular survival, etc.)?
- What is the order of secretion of effector proteins and how is export regulated?
- What are the host cell targets of the effector proteins?
- How do effector proteins regulate host cell signaling pathway(s)?
- Which effector proteins are unique to clinical isolates versus commensal isolates?
- How do the effector proteins modulate the environmental niches and clinical manifestations?

## Data Availability

Not applicable.

## Supplementary Materials

The following supporting information can be downloaded at: https://www.mdpi.com/article/10.3390/biom13010135/s1, Figure S1: Pie chart of Clusters of Orthologous Genes (COG) functional categories of *C. jejuni* flagellar type III secreted proteins predicted by the SIEVE algorithm; Table S1: *Campylobacter* and *Salmonella* flagellar proteins; Table S2: *C. jejuni* type III secreted proteins predicted by the SIEVE algorithm [79,113,114,115,117,125,130,140,143,144,145,146,147,148,149,164,239,240,241,242,243,244,245,246,247,248,249,250,251,252,253,254,255,256,257,258]; Table S3: Components of the type IV secretion system in *C. jejuni*; Table S4: Components of the type VI secretion system in *C. jejuni*.
