# Supplementary material for: The Missing Pieces: The Role of Secretion Systems in Campylobacter jejuni Virulence
Source: Biomolecules. 2023 Jan 9;13(1):135. doi: 10.3390/biom13010135 (PMC9856085; doi:10.3390/biom13010135)

Figure S1. Pie chart of Clusters of Orthologous Genes (COG) functional categories of *C. jejuni* flagellar type III secreted proteins predicted by the SIEVE algorithm. Only proteins that have the SIEVE scores of above 1.3 are considered for these categories. About 60% (n=34) of secreted proteins fall into known COG categories. At least seven proteins are involved in cell motility, five have a role in cell wall/membrane biogenesis, and four have a general function. Six proteins do not have any assigned COG categories. About 30% (n=17) of proteins do not have any assigned COG categories but have ENOG numbers EggNOG v6.0 in Uniprot database.

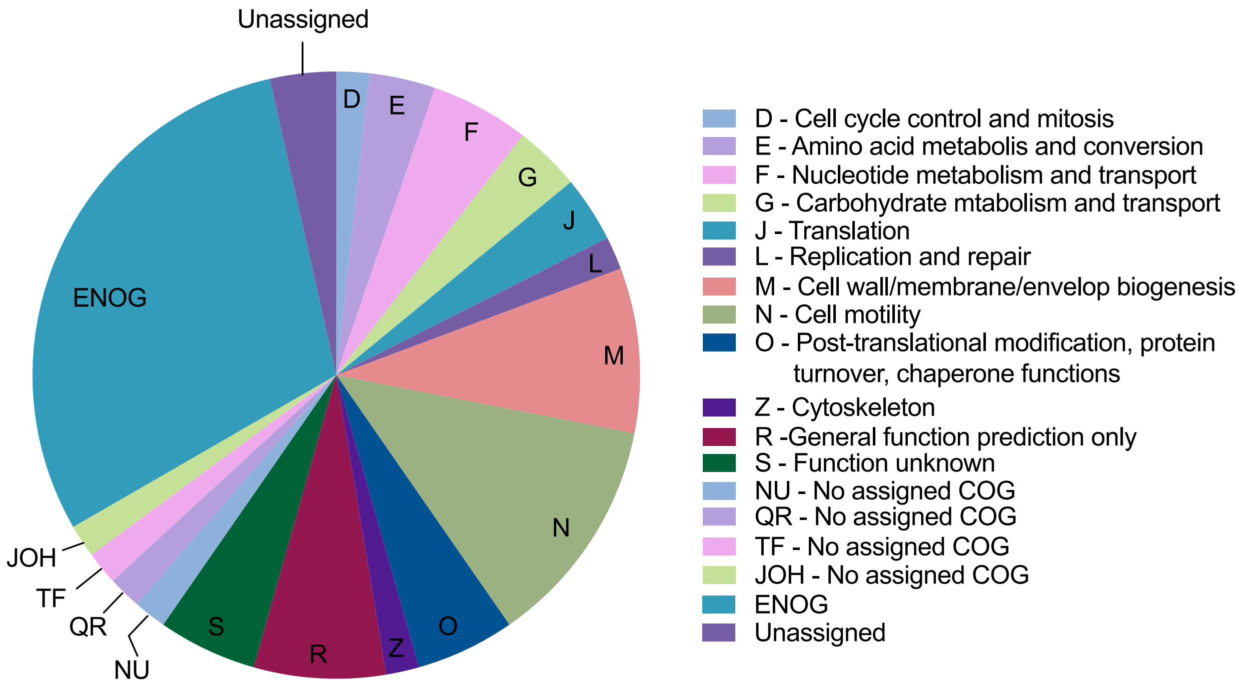

Supplement: Supplementary file 1 [file biomolecules-13-00135-s001.zip › Figure S1.zip!/Figure S1.pdf]
