# Supplementary material for: The Missing Pieces: The Role of Secretion Systems in Campylobacter jejuni Virulence
Source: Biomolecules. 2023 Jan 9;13(1):135. doi: 10.3390/biom13010135 (PMC9856085; doi:10.3390/biom13010135)
Supplement: Supplementary file 1 [file biomolecules-13-00135-s001.zip › z3 Table S3 landscape 12-15.zip!/z3 Table S3 landscape 12-15.pdf]

STable 3. *Campylobacter jejuni* relationship to *Helicobacter pylori* Cag T4SS genes.

Protein	Function	*C. jejuni* strain 81-176				% Present in *Campylobacter* Genomes[1]
		Protein Identity	Protein Coverage	pTet	pVir	
Cagz/Cag1	Membrane-associated protein; potential influence CagA translocation	0%	0%	No Match	No Match	0.00%
Cage/Cag2		0%	0%	No Match	No Match	0.00%
Cagd/Cag3	Outer membrane complex	0%	0%	No Match	No Match	0.00%
Cagg/Cag4/VirB1	Transglycosylase, peptidoglycan hydrolase, t-pilus formation	0%	0%	No Match	No Match	0.00%
Cagb/Cag5/VirD4	ATPase, coupling protein, recruitment of substrate to T4SS channel	31.20%	79.81%	Cjj81176_pTet0040	Cjj81176_pVir0007	32.62%
Caga/VirB11	ATPase	44.60%	88.48%	Cjj81176_pTet0039	Cjj81176_pVir0005	33.35%
CagA	Effector protein	0%	0%	No Match	No Match	0.00%
CagB		0%	0%	No Match	No Match	0.00%
CagC	Major subunit of extracellular pilus	0%	0%	No Match	No Match	0.00%
CagD		0%	0%	No Match	No Match	0.00%
CagE	ATPase	31.40%	82.60%	Cjj81176_pTet0028	Cjj81176_pVir0053	32.81%
CagF	Secretion chaperone	0%	0%	No Match	No Match	0.00%
CagG		0%	0%	No Match	No Match	0.00%
CagH		0%	0%	No Match	No Match	0.00%
CagI		0%	0%	No Match	No Match	0.00%
CagL/VirB5	Minor subunit of extracellular pilus, pilus tip adhesion, Integrin binding	0%	0%	No Match	No Match	0.00%
CagM	Outer membrane complex	0%	0%	No Match	No Match	0.00%
CagN		0%	0%	No Match	No Match	0.00%
CagP		0%	0%	No Match	No Match	0.00%
CagQ		0%	0%	No Match	No Match	0.00%
CagS		0%	0%	No Match	No Match	0.00%
CagT/VirB7	Pore composition, Binds to VirB9 and stabilizes it	30.40%	40.00%	Cjj81176_pTet0041	No Match	3.57%
CagU		0%	0%	No Match	No Match	0.00%
CagV/VirB8	Biopic membrane protein, core complex	26.70%	69.05%	Cjj81176_pTet0036	Cjj81176_pVir0001	24.21%
CagW/VirB9	Polytopic membrane protein, stabilization of VirB5 and VirB3, inner membrane channel	0%	0%	No Match	No Match	0.00%
CagX/VirB9	Pore composition, intermolecular disulfide bridge with VirB7	27.0%	44.15%	Cjj81176_pTet0037	Cjj81176_pVir0002	25.20%
CagY/VirB10	Biopic membrane protein, Scaffolding of central pore channel. Pilus production. ATP energy sensor	29.80%	11.21%	Cjj81176_pTet0038	Cjj81176_pVir0003	32.51%
CagZ	Cagb stabilization	0%	0%	No Match	No Match	0.00%

[1]Percentage of hits based on screening
