# Supplementary material for: The Missing Pieces: The Role of Secretion Systems in Campylobacter jejuni Virulence
Source: Biomolecules. 2023 Jan 9;13(1):135. doi: 10.3390/biom13010135 (PMC9856085; doi:10.3390/biom13010135)
Supplement: Supplementary file 1 [file biomolecules-13-00135-s001.zip › Z4 Supplemental Table S4.zip!/Z4 Supplemental Table S4.pdf]

Supplemental Table S4. *Vibrio cholerae* T6SS structural genes and *C. jejuni* homologs

***V. cholerae* T6SS Gene**	**T6SS Gene**	**Function/Location**	***C. jejuni* str. M129**			**% Present in *Campylobacter* Genomes[1]**
			**Protein Identity**	**Protein Coverage**	**Locus Tag**	
TssA	VasJ/ImpA	Baseplate complex recruiter, Polymerization of TssD with sheath	20.2%	74.41%	CJM129_4895	22.33%
TssB	VipA/ImpB	Contractile sheath	50.6%	91.67%	CJM129_4900	24.59%
TssC	VipB/ImpC	Contractile sheath	60.3%	96.33%	CJM129_4905	24.71%
TssD	Hcp	Secreted tube, effector	63.3%	67.67%	CJM129_4875	24.84%
TssE	HsiF	Baseplate complex	24.2%	62.76%	CJM129_4910	24.38%
TssF	VasA/ImpG	Baseplate complex	30.4%	96.77%	CJM129_4915	24.25%
TssG	VasB/ImpH	Baseplate complex	26.2%	92.31%	CJM129_4920	23.77%
TssH	ClpV/VasG	AAA+ATPase, sheath recycling	40.9%	69.74%	CJM129_2355*	99.74%*
TssI	VgrG	Spike, effector	24.0%	48.12%	CJM129_5040	26.78%
TssJ	VasD/Lip/SciN	Membrane complex	0.0%	0.0%	N/A	0.00%
TssK	VasE/ImpJ	Baseplate	24.4%	98.42%	CJM129_4885	23.74%
TssL	IcmH/DotU/VasF	Membrane complex	29.7%	31.86%	CJM129_4880	23.80%
TssM	IcmF/VasK	Membrane complex	22.7%	40.39%	CJM129_4870	21.59%
TagD	PAAR	Tip of spike	0.0%	0.0%	N/A	0.06%

*Note: Hit is another ATPase (ClpB protein) not present in the CJIE3, but located elsewhere on the chromosome
[1]Percentage of matches based on screening PubMLST database
